# A Simple Algorithm for Population Classification

**DOI:** 10.1038/srep23491

**Published:** 2016-03-31

**Authors:** Peng Hu, Ming-Hua Hsieh, Ming-Jie Lei, Bin Cui, Sung-Kay Chiu, Chi-Meng Tzeng

**Affiliations:** 1 Translational Medicine Research Center (TMRC), School of Pharmaceutical Science, Xiamen University, Xiamen P.R. China; 2 Key Laboratory for Cancer T-Cell Theranostics and Clinical Translation (CTCTCT), Xiamen P.R. China; 3 Department of Risk Management and Insurance, National Chengchi University, Taiwan; 4 Department of Endocrine and Metabolic Diseases, Rui-jin Hospital, Shanghai Jiao-tong University School of Medicine, Shanghai 200025, China; 5 Department of Biology and Chemistry, City University of Hong-Kong, Hong Kong

## Abstract

A single-nucleotide polymorphism (SNP) is a variation in the DNA sequence that occurs when a single nucleotide in the genome differs across members of the same species. Variations in the DNA sequences of humans are associated with human diseases. This makes SNPs as a key to open up the door of personalized medicine. SNP(s) can also be used for human identification and forensic applications. Compared to short tandem repeat (STR) loci, SNPs have much lower statistical testing power for individual recognition due to the fact that there are only 3 possible genotypes for each SNP marker, but it may provide sufficient information to identify the population to which a certain samples may belong. In this report, using eight SNP markers for 641 samples, we performed a standard statistical classification procedure and found that 86% of the samples could be classified accurately under a two-population model. This study suggests the potential use of SNP(s) in population classification with a small number (n ≤ 8) of genetic markers for forensic screening, biodiversity and disaster victim controlling.

Since the single nucleotide polymorphisms (SNPs) are genetic variations which determine the difference across members of the same species, the SNPs can be used to identify the correct source population of an individual. In recent years there have been several publications about the application of SNP technology in the forensic, human identification and population classification^1^,^2^,^3^,^4^. Such as Nina Zhou *et al.*^1^, using a ranking measure, i.e., a modified *t*-test or F-statistics, combined with the support vector machine (SVM) classifier, they had found that using on average 64 SNPs could obtain 82.46 ± 11.41% classification accuracy for 3 population classification. In another studies, Kohnemann *et al.*^4^ showed a potential application of a mitochondrial DNA (mtDNA) SNP analysis for forensic application, 32 SNPs were detected in a multiplex polymerase chain reaction (PCR) assay and a multiplex SNaPshot analysis. In the analysis cases, STR-analysis and sequencing of the mtDNA hyper-variable region I (HVR I) failed and the mtDNA SNP analysis was the only way to obtain satisfactory results, even in a case with mixed stains. Yet, all these analyses are time-consuming and very expensive, since if we want to ensure the accuracy of the classification procedure and obtain a desirable feature subset SNPs with the minimum size and most informativeness, the number of SNP must be at least 30–70 in all these analyses^5^,^5^,^5^,^5^. Nevertheless, when applied to appropriate data set, data mining and machine learning techniques can be more effective on feature selection and therefore provide excellent classification accuracy. Sushmita Mitra *et al.*^5^ had made a positive summary about various machine learning techniques, a.k.a. soft computing in bioinformatics. For example, a gene is a long DNA sequence so that each gene is much more powerful than a SNP for classification. In light of this difference between a gene and a SNP, Lipo Wang *et al.* had done excellent works^6^,^7^,^8^, providing effective methods of gene selection and finding that just a few genes can give very accurate cancer classification. In particular, they use SVM and Fuzzy Neural Network (FNN) to find the minimum gene subset after the step of gene importance ranking^6^. They find 2 genes are sufficient to produce high classification accuracy. Another work is based on spectral biclustering to find just 2 genes and provide 99.92% classification accuracy for Lymphoma; and just one gene to produce 98.7% classification accuracy for liver cancer^7^. In the other work, they provide effective method for gene selection in high dimensional data, such as microarray gene expression data^8^. Basically, they apply dimensional reduction technique first and then apply a voting scheme by utilizing binary SVMs. They found t-test-based gene selection is most effective among others. For SRBCT and lymphoma data set, they can use just 6 and 5 genes to give a 100% accurate classification. In this report, we adopted the simple extension of the standard likelihood ratio test and performed a standard statistical classification procedure to minimum the number of the SNP and enhance the classification accuracy. Those could be applied either in forensics or in disaster control precisely and promptly.

## Problem Statement and Classification Procedure

Suppose there are two human populations, A and B, which are in accordance with Hardy-Weinberg equilibrium. Populations A and B have different frequencies of SNP genotypes, and there is no linkage disequilibrium between SNP sites. Each genotype of a given SNP (i) follows a trinomial distribution with parameters *p*_*i*_, *q*_*i*_, and *r*_*i*_ (*p*_*i*_ + *q*_*i*_ + *r*_*i*_ = 1) in population A and each genotype of the same given SNP (i) follows a trinomial distribution with parameters *u*_*i*_, *v*_*i*_, and *w*_*i*_ (*u*_*i*_ + *v*_*i*_ + *w*_*i*_ = 1) in population B. In particular, if the genotypes of SNP (i) consist of the data set {CC, CT, TT}, then a random selected individual from population A has the following properties:

1. Prob (genotype of SNP (i) = CC) = *p*_*i*_
2. Prob (genotype of SNP (i) = CT) = *q*_*i*_
3. Prob (genotype of SNP (i) = TT) = *r*_*i*_

If the individual is from population *B*, then the following is true:

- 4. Prob (genotype of SNP (i) = CC) = *u*_*i*_
- 5. Prob (genotype of SNP (i) = CT) = *v*_*i*_
- 6. Prob (genotype of SNP (i) = TT) = *w*_*i*_

When the genotypes of SNP(i) consist of the data set {AA, AG, GG}, the probability statements are the same as above except CC, CT, and TT are replaced by AA, AG, and GG, respectively.

Given an individual sample *S* consisting of *n* SNP markers, the problem is to determine whether the individual comes from population *A* or *B*. The likelihood functions of *S* must be derived.

L(*A*) = likelihood function (*S* is from population *A*)

7. 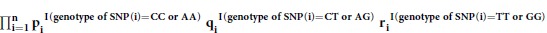


L(*B*) = likelihood function (*S* is from population *B*)

8. 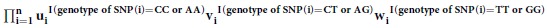


Where I(.) is the indicator function.

A simple classification procedure can then be defined based on likelihood functions L (*A*) and L (*B*):

If L (*A*)/L (*B*) > 1, the individual is from population *A*. Otherwise, the individual is from population *B.* The model we proposed is in a general setting. A population can be any specific group of people and population parameters can be estimated from representative samples. For example, in this paper, Population A is the group of people lives in a specific geographical region and Population B is the general population. Parameters of Population A and B are estimated using representative samples from an epidemic society of Shanghai and NCBI, respectively. Such application setting is common for life insurers. For example, to design and price a medical insurance contract of a 65-years-old male, actuary needs to analyze two populations: Populations A represents 65-years-old male specific to the life insurer, due to the company screening process for the policy holders and Population B represents all of 65-years-old males. Representative samples of Population A and B then come from the internal database of the insurer and public organizations such as National Association of Insurance Commissioners (NAIC) or Society of Actuaries (SOA), respectively. The population parameters estimated by representative samples may entail some estimation errors. However, as we are entering the era of “Big Data”, the representative samples are converging to the true populations.

The algorithm is a simple extension of the standard likelihood ratio test based on Neyman-Pearson lemma^9^. The statistical efficiency depends on the difference between parameters of two populations and the number of SNPs used to calculate the likelihood functions. We investigate the empirical efficiency of this algorithm based on 8 selected SNPs in Section 4. The standard likelihood ratio test assumes the selected individual come from A or B population with equal probability. This assumption is suitable for general statistical hypothesis tests. However, if the population sizes are known and the individual is a random sample from these two populations, then it would be more appropriate to incorporate the population size into consideration. Considering this, the algorithm also could be presented as: Suppose there are two populations: population A with size N (A), and population B with size N (B). There is a sample with genotype (G). Let’s L(X) be the likelihood function defined in the manuscript. Let E (A) = N (A) × L (A). Therefore E (A) is the expected number of samples (with genotype G) found in population A. E (B) is the same for population B. It is then appropriate to use the ratio E (A)/E (B) to determine the origin of this sample.

## Eight-Marker SNP Sample from a Chinese Population

Based on the information from the Human Genome and International HapMap Projects^10^, eight SNP markers having a high allele mutation frequency (0.249 < MAF < 0.355), were randomly selected from eight different chromosomes, respectively: rs2243191 (1q32), rs2856838 (2q14), rs583911 (3q25), rs2227306 (4q13), rs20541 (5q31), rs8193036 (6p12), rs4739139 (8q12), and rs741344 (12q15). A sample of 641 was collected from an epidemic society of Shanghai. The eight SNPs were selected from HapMap, which was with high allele mutation frequency from random selected SNPs in human being. After filtering by minimum allele frequency (MAF), the MAF of the eight SNPs is between 0.249and 0.355, which basically meet the experimental set P ≈ 0.333. Therefore, we could take advantage of higher equilibrium factor to group the independent classifications. SNPs could be picked as randomly, but with higher frequency in species.

All extracted gDNA by MagCore HF-16 (RBC Bioscience) was subjected to quality control using a threshold of 260/280 ratio and validated with a final concentration of 10 ng/ul. SNPstream (Beckman Coulter) was used for SNP genotyping in this study. Primers were designed and generated using Autoprimer (http://www.autoprimer.com/). PCR amplification, amplicon purification, DNA hybridization, and data analysis were accomplished using an SNP stream automation analyzer.

Haploview provided a summary table for the SNP sample^11^. The characteristics of these eight SNP markers are summarized in Table 1, they are located on different chromosomes. The *P*-values from Hardy-Weinberg equilibrium analysis of each of the markers were all greater than 0.05. This indicates these alleles are in equilibrium^12^.

This classification procedure is based on the frequencies (0.249 < MAF < 0.355) of genotypes of SNPs. Their frequencies are summarized in Table 2. This population played the role of population *A* in the classification procedure.

## Frequencies of SNP markers from the NCBI SNP database

To determine the classification procedure defined in Section 1, we determined the genotype frequencies of the SNPs from the NCBI SNP database at http://www.ncbi.nlm.nih.gov/SNP/index.html. The collected information is summarized in Table 3. The last row of Table 3 shows the number of individuals used to compute the genotype frequencies of each SNP marker. We treated the genotype frequencies of SNP markers as having been from a “general” population. This population played the role of population *B* in the classification procedure.

In our hypothetical situation, the hypothetical suspect “S” comes from population A, it is natural to set the general population of Chinese as population B. If there are two suspects, it would be natural to set population A & B as their origins, respectively.

## Accuracy of the classification procedure

We tested the classification procedure using the frequency data described in Sections 2 and 3. To determine the impact of the number of SNP markers on the accuracy of the classification procedure, we set the number of SNP markers *n* = 2, 4, 6, and 8. The accuracy of the classification depends on the multinomial parameters of the two populations, such as population size, the difference between two populations and the selection and number of SNPs. It is common to use the power of the test to represent the accuracy of classification. If the parameters of the two populations were entirely different, then the likelihood ratio in the algorithm would converge to infinity quickly. In such situation, the power of the test is high. In terms of classification accuracy, increasing the numbers of SNPs will only increase the power (accuracy). The results are shown in Table 4. It became clear that accuracy increased when the number of SNP markers used was increased. when n ≥ 8, such as 10–12 ,the accuracy is saturated around 88–90%(data not shown) without increasing significantly. The results indicate that a sample with a smaller number of SNP markers can be useful in identifying the population from which a given individual may have come.

## Discussion

With the human genome project and haplotype-depth research program, SNP genotyping has been applied in disease diagnosis, population genetics, pharmacogenomics, and many other fields. The development of restriction fragment length polymorphism (RFLP) and short tandem repeat microsatellite markers (STR) have led to widespread use of SNPs in many types of applications.

The most successful application of SNP detection is in the field of forensic genetics, where it is used to evaluate rare, degraded, and even nearly fossilized nucleic acid evidence. It has also been used in the identification of human beings, animals, and goods and in the study of race, migration, evolution, lineage, and intellectual property issues. FFPE samples from clinics and universities could be used in SNP analysis to decipher genetic markers relevant to risk assessment, prognosis, and therapeutic diagnosis^13^,^14^,^15^. However, SNP is restricted by the fact that there are only three possible polymorphisms per residue. The identification power of the number of SNPs is about 50–70 rather than 13 for STRs^16^, to core the populatin of the world.

The purpose of this study is to take advantage of the efficiency and simplicity of SNP detection to process data from large populations and to reduce the number of SNP targets. STR is restricted by assigned primers and restriction enzyme. SNP pools can be easily adjusted based on the frequency and identity of the mutation in question. We here propose a statistical algorithm for empirical SNP detection to increase the power of classification and to narrow down the factors for criminal screening using this effective method.

## Additional Information

**How to cite this article**: Hu, P. *et al.* A Simple Algorithm for Population Classification. *Sci. Rep.* **6**, 23491; doi: 10.1038/srep23491 (2016).

## Figures and Tables

**Table 1:** Haploview summary.

Marker	Position	ObsHET	HWpval	MAF	Allele
rs2243191	01q32	0.406	0.8058	0.276	T:C
rs2856838	02q14	0.379	0.8236	0.249	C:T
rs583911	03q25	0.412	0.2625	0.268	G:A
rs2227306	04q13	0.476	0.3764	0.355	C:T
rs20541	05q31	0.426	0.9762	0.310	C:T
rs8193036	06p12	0.407	0.7335	0.293	C:T
rs4739139	08q12	0.445	0.0505	0.289	C:T
rs741344	12q15	0.465	0.4259	0.340	A:G

**Table 2:** Frequencies of genotypes of SNP markers in population *A.*

	rs2856838	rs8193036	rs2243191	rs20541	rs2227306	rs4739139	rs741344	rs583911
CC	0.562	0.504	0.073	0.477	0.407	0.488	0.000	0.000
CT	0.379	0.407	0.406	0.426	0.476	0.445	0.000	0.000
TT	0.059	0.089	0.521	0.097	0.117	0.067	0.000	0.000
AA	0.000	0.000	0.000	0.000	0.000	0.000	0.427	0.062
AG	0.000	0.000	0.000	0.000	0.000	0.000	0.465	0.412
GG	0.000	0.000	0.000	0.000	0.000	0.000	0.108	0.526

**Table 3:** Frequencies of genotypes of SNP markers in population *B.*

	rs2856838	rs8193036	rs2243191	rs20541	rs2227306	rs4739139	rs741344	rs583911
CC	0.460	0.159	0.480	0.603	0.567	0.733	0.000	0.000
TC	0.399	0.433	0.353	0.331	0.361	0.242	0.000	0.000
TT	0.141	0.409	0.168	0.066	0.072	0.025	0.000	0.000
AA	0.000	0.000	0.000	0.000	0.000	0.000	0.437	0.377
GA	0.000	0.000	0.000	0.000	0.000	0.000	0.425	0.358
GG	0.000	0.000	0.000	0.000	0.000	0.000	0.139	0.264
Count	652	1817	1934	1247	610	554	504	1468

**Table 4:** Frequencies of genotypes of SNP markers.

SNP	rs2856838	rs2856838	rs2856838	rs2856838
Markers	rs8193036	rs8193036	rs8193036	rs8193036
		rs2243191	rs2243191	rs2243191
		rs20541	rs20541	rs20541
			rs2227306	rs2227306
			rs4739139	rs4739139
				rs741344
				rs583911
Accuracy	73.9%	81.7%	83.0%	86.0%
